# Association of Serum Carotenoid Concentration and Dietary Habits among the JACC Study Subjects

**DOI:** 10.2188/jea.15.S220

**Published:** 2005-08-18

**Authors:** Kotaro Ozasa, Yoshinori Ito, Koji Suzuki, Yoshiyuki Watanabe, Kenji Wakai, Akiko Tamakoshi

**Affiliations:** 1 Department of Epidemiology for Community Health and Medicine, Kyoto Prefectural University of Medicine Graduate School of Medical Science.; 2 Department of Public Health, Fujita Health University School of Health Sciences.; 3 Division of Epidemiology and Prevention, Aichi Cancer Center Research Institute.; 4 Department of Preventive Medicine/Biostatistics and Medical Decision Making, Nagoya University Graduate School of Medicine.

**Keywords:** Carotenoids, Food Habits, validity, Cohort Studies

## Abstract

BACKGROUND: We wished to determine the validity of the association between serum carotenoid concentrations and dietary habits obtained from a food frequency questionnaire in the Japan Collaborative Cohort Study (JACC Study) for Evaluation of Cancer Risk sponsored by the Ministry of Education, Science, Sports and Culture of Japan (Monbusho).

METHODS: The subjects were 866 male and 569 female controls in nested case-control studies for evaluating the risk of lung, colorectal, and urothelial cancers as parts of the JACC Study. Dietary habits were assessed using a food frequency questionnaire, and serum samples were obtained at baseline. Serum carotenoid concentrations of frozen-stored sera were measured and compared with the results of the survey.

RESULTS: In males, consumption of dairy products, some oily foods, vegetables, fruits, and boiled beans correlated positively with serum carotenoid concentrations, whereas ingestion of boiled rice and *sansai* (edible wild plants) was negatively correlated with serum carotenoids. In females, only fruit consumption was positively correlated with serum carotenoid concentration, whereas ingestion of butter, *sansai*, and potatoes were negatively correlated. Some specific associations, between serum lycopene and tomato consumption and between serum *β*-cryptoxanthin and ingestion of oranges, were observed in both sexes.

CONCLUSIONS: In males, serum carotenoid concentrations were slightly associated with intake of foods rich in carotenoids. The lack of associations in females suggests that the food frequency questionnaire did not validly evaluate females’ dietary habits concerning carotenoids in the JACC Study.

Carotenoids are contained in various vegetables and fruits, especially those colored deep green, yellow, or red. The concentration of some carotenoids is especially high in certain vegetables or fruits, for example, lycopene in tomatoes and cryptoxanthin in oranges.^1^ Carotenoids are thought to have anti-carcinogenic properties, which are associated with their anti-oxidant activity as well as other mechanisms.^2^ Studies in cancer epidemiology have revealed associations between carotenoid intake or serum concentration and cancer risk.

Epidemiologic studies of the risk of chronic diseases such as cancer typically evaluate the risks associated with long-term, stable lifestyles. Many of these studies use questionnaires to determine the average conditions over long periods of time prior to having the target illness. Serum concentrations of nutrients or other constituents at baseline are not good determinants of the average concentration over time, but are merely snapshots taken at a single time point. For example, data from a single ingestion of *β*-carotene showed that its half life was about 10 days,^3^ suggesting that serum carotenoid concentrations reflect their intake in daily life. To assess the validity of questionnaire surveys, however, it is necessary to compare their results with those from serum measurements.^4^^-^^6^

During the Japan Collaborative Cohort Study (JACC Study) for Evaluation of Cancer Risk sponsored by the Ministry of Education, Science, Sports and Culture of Japan (Monbusho), a food frequency questionnaire was used to assess dietary habits of the subjects at baseline survey. In addition, blood samples were collected from all subjects at baseline,^7^ and serum concentrations of carotenoids were measured to evaluate cancer risk at lung,^8^ colon and rectum,^9^ and urothelial system (pelvis, ureter, and bladder).^10^ To validate these results, we have compared serum carotenoid concentrations with dietary habits in a sample set of the JACC Study.

## METHODS

The subjects of this study were 866 male (mean ± standard deviation of age, 64.4 ± 8.3 years) and 569 female (mean ± SD of age, 64.0 ± 8.3 years) controls in nested case-control studies, conducted as parts of the JACC Study, which examined the risks of lung, colorectal, and urothelial cancers related to serum carotenoid concentrations. Their dietary habits were surveyed from 1988 to 1990 using a self-administered food frequency questionnaire, in which the subjects indicated their average consumption of 32 food items (beef, pork, ham and sausage, chicken, liver, egg, milk, yogurt, cheese, butter, margarine, fried food, fried vegetables, fish (unprocessed), boiled fish paste (‘*kamaboko*’ in Japanese), dried or salted fish, green-leaf vegetables, carrots and squash, tomatoes, cabbage and lettuce, Chinese cabbage, edible wild plants (‘*sansai*’), mushroom, potatoes, seaweed, pickles, food boiled down in soy sauce (‘*tsukudani*’), boiled beans, tofu, oranges, fruits other than oranges, and fruit juice). Subjects indicated frequency of consumption as scarcely, 1-2 times a month, 1-2 times a week, 3-4 times a week, and almost every day. Boiled rice was evaluated as the number of bowls consumed per day. Consumption of miso soup was evaluated as scarcely, a few times per month, every other day, almost every day, with the latter indicating the number of bowls consumed.

The subjects donated blood samples at health-screening checks sponsored by municipalities during the same time period as the questionnaire survey. As soon as possible after blood drawing, serum was separated at laboratories in or near the municipalities and stored at -80°C until analyzed. Individual written or oral consent, or consent from community representatives, was obtained for the subjects, or a poster notification/opting-out system was applied.^7^ The Ethical Boards of the Nagoya University School of Medicine, Fujita Health University, and the Kyoto Prefectural University of Medicine approved this study.

We assayed serum concentrations of carotenoids by high-performance liquid chromatography (HPLC).^11^ Because we did not separately measure serum zeaxanthin and lutein, we recorded their combined levels as zeaxanthin/lutein. The total carotene concentration was recorded as the sum of a *α*- and *β*-carotenes and lycopene; total xanthophyll concentration was recorded as the sum of *β*-cryptoxanthin, canthaxanthin and zeaxanthin/lutein. Total carotenoid concentration was calculated as total carotenes plus total xanthophylls. Serum concentrations of total cholesterol were assayed at the SRL Laboratory (Hachioji, Japan) in 2001-2003.

Associations between serum carotenoid concentrations and lifestyle, including dietary habits (frequency of food consumption), smoking, and drinking, were evaluated using Spearman’s correlation coefficients (*r_s_*). Missing values for smoking and drinking were treated as an additional category or replaced with surrogate values to determine the rank used for adjustment. Subjects who did not answer the question whether they were smoking, quit smoking, or did not smoke were treated as a category of ‘unknown smoking status.’ Their serum carotenoid concentrations were similar to those of non- or ex-smokers. For current smokers who did not indicate the number of cigarettes consumed per day, we assumed that males smoked 18.9 and females smoked 11.4 cigarettes, the mean numbers of cigarettes smoked per day by each sex. Consequently, smoking was ranked in the order non-smokers, unknown smoking status, ex-smokers, and current smokers, with the latter ranked by number of cigarettes consumed per day. Of the 866 males, 396 were current smokers, 247 were ex-smokers, 192 were nonsmokers, and 31 were of unknown smoking status. Of the 569 females, 16 were current smokers, 14 were ex-smokers, 475 were nonsmokers, and 64 were of unknown smoking status.

Alcohol drinking was treated in the same manner, with current drinkers who did not indicate the amount of alcohol consumed per day assigned their respective gender means (45 mL for males and 17 mL for females). Of the 866 males, 604 were current drinkers, 39 were ex-drinkers, 193 were nondrinkers, and 30 were of unknown drinking status. Of the 569 females, 101 were current drinkers, 7 were ex-drinkers, 419 were nondrinkers, and 42 were of unknown drinking status.

## RESULTS

The geometric means of serum carotenoid concentrations and the back-transformed values of log-transformed mean ± standard deviation are shown by sex in Table 1. These concentrations were generally higher in females. When we segregated serum carotenoid concentrations by age, smoking, and drinking status, we found that some carotenoids were sporadically correlated, either positively or negatively, with age in both sexes. Almost all the carotenoids were negatively correlated with smoking and drinking in males, but not in females. Serum concentrations of carotenoids and total cholesterol were correlated in both sexes; the range of *r_s_* was 0.23 to 0.34 for males and 0.19 to 0.38 for females.

**Table 1.** Distribution of serum concentrations of carotenoids and vitamins, and Spearman’s correlation coefficients with age and smoking.

	Geometric means (µM) and means ± SD^†^				Spearman’s correlation coefficients					
					Age		Smoking ^‡^		Alcohol drinking ^‡^	
			
	Males (n=866)		Females (n=569)		Males	Females	Males	Females	Males	Females
Zeaxanthin/lutein	0.91	0.52, 1.60	1.02	0.59, 1.76	0.04	-0.00	-0.08**	-0.03	0.01	-0.02
Canthaxanthin	0.03	0.01, 0.05	0.03	0.02, 0.05	0.01	-0.09*	-0.06+	-0.04	-0.06*	0.00
*β*-Cryptoxanthin	0.17	0.06, 0.50	0.32	0.13, 0.81	-0.02	-0.08^+^	-0.15**	-0.02	-0.17**	0.05
Total xantophylls	1.18	0.66, 2.12	1.46	0.83, 2.57	0.02	-0.02	-0.12**	0.01	-0.04	0.00
*α*-Carotene	0.05	0.02, 0.13	0.09	0.04, 0.19	0.02	-0.10*	-0.13**	-0.00	-0.17**	0.04
*β*-Carotene	0.32	0.11, 0.97	0.64	0.26, 1.55	0.08*	-0.03	-0.17**	-0.04	-0.21**	0.00
Lycopene	0.11	0.04, 0.33	0.20	0.08, 0.52	-0.06*	-0.06	-0.08*	-0.08*	-0.11**	0.01
Total carotenes	0.53	0.20, 1.42	1.00	0.45, 2.22	0.04	-0.06	-0.17**	-0.05	-0.20**	0.01
Total carotenoids	1.78	0.92, 3.46	2.54	1.38, 4.68	0.03	-0.04	-0.16**	-0.01	-0.12**	0.01

** p<0.01, * p<0.05, + p<0.1

† : Backtransformation from log-transformed data

‡ : Smoking and drinking were ranked, and missing numbers were supplied, as described in Methods part.

The association of serum concentrations of total carotenoids and the frequency of intake of individual food items is shown in Table 2. In males, ingestion of eggs, dairy products, some oily foods, vegetables, fruits, and boiled beans were positively correlated with serum concentrations of carotenoids, whereas boiled rice and sansai were negatively correlated. In females, only intake of fruits was positively correlated to serum concentration of total carotenoids, whereas intake of butter, *sansai*, and potatoes was negatively correlated. These tendencies remained after adjusting for age, smoking, drinking, and serum concentration of total cholesterol (Table 2). After further adjustment for intake of oranges, other fruits, green-leafy vegetables, carrots and squash, and tomatoes, the positive correlation of serum carotenoid concentration with intake of eggs and milk, and the negative correlation with intake of *sansai*, still remained in males. In females, however, there were negative correlations between serum carotenoid concentration and the intake of butter, fish, dried and salted fish, and potatoes.

**Table 2.** Spearman’s correlation coefficients of foods with total carotenoids and smoking.

	Total carotenoids^†^		Total carotenoids^‡^		Total carotenoids^§^		Smoking^†^	
			
	Males	Females	Males	Females	Males	Females	Males	Females
Boiled rice	-0.11**	-0.04	-0.07*	-0.04	-0.04	-0.03	-0.00	-0.07^+^
Miso soup	-0.06^+^	-0.06	-0.00	-0.03	-0.05	-0.02	-0.00	-0.13**
Beef	-0.02	-0.04	-0.02	-0.07	-0.02	-0.10^+^	0.06^+^	0.03
Pork	0.03	0.00	0.05	0.00	0.00	0.01	0.04	-0.08^+^
Ham and sausage	0.04	-0.05	0.04	-0.08^+^	0.00	-0.05	-0.02	-0.05
Chicken	-0.02	0.05	-0.00	-0.01	-0.06	-0.02	-0.05	-0.08^+^
Liver	-0.02	-0.02	0.01	-0.00	-0.01	-0.03	0.01	0.09^+^
Eggs	0.12**	-0.00	0.12**	0.01	0.10*	-0.03	-0.07*	-0.06
Milk	0.27**	0.07	0.20**	0.04	0.16**	0.05	-0.10**	-0.05
Yogurt	0.15**	0.03	0.10*	0.00	0.04	0.02	-0.06^+^	0.05
Cheese	0.12**	0.01	0.11**	-0.00	0.06	-0.00	-0.02	0.04
Butter	0.07^+^	-0.12**	0.08*	-0.12*	0.04	-0.15**	0.00	-0.04
Margarine	0.08*	0.01	0.03	0.01	-0.01	0.00	-0.04	0.01
Fried foods	0.07*	-0.03	0.06	-0.02	0.06	-0.05	-0.08*	-0.13**
Fried vegetables	0.03	0.00	0.02	0.03	0.00	0.03	-0.13**	-0.06
Fish (unprocessed)	-0.00	-0.05	-0.01	-0.08^+^	-0.06	-0.14**	-0.04	-0.09*
*Kamaboko* ^#^	0.06	-0.03	0.05	-0.00	0.00	-0.00	-0.05	-0.05
Dried or salted fish	0.02	-0.05	0.04	-0.05	-0.00	-0.11*	0.05	-0.09*
Green-leafy vegetables	0.06^+^	0.00	0.07^+^	0.02	-	-	-0.11**	-0.01
Carrots and squash	0.07*	0.00	0.08*	-0.01	-	-	-0.08*	-0.15**
Tomatoes	0.08*	0.05	0.10**	0.02	-	-	-0.05	-0.14**
Cabbage and lettuce	0.07*	-0.01	0.02	-0.02	-0.06	-0.03	-0.06^+^	-0.03
Chinese cabbage	0.08*	-0.04	0.09*	-0.01	0.00	-0.02	-0.06^+^	0.03
Sansai^¶^	-0.12**	-0.10*	-0.10**	-0.07	-0.10*	-0.09^+^	-0.01	-0.01
Mushrooms	0.01	-0.01	0.01	0.01	-0.03	-0.01	0.00	0.00
Potatoes	0.03	-0.10*	0.01	-0.09*	-0.06	-0.13*	-0.08*	-0.13**
Seaweeds	0.03	0.01	0.06	0.02	-0.04	0.03	0.00	-0.03
Pickles	-0.05	0.01	-0.02	0.04	-0.02	0.05	0.04	-0.06
*Tsukudani* ^† †^	-0.01	-0.02	0.00	-0.00	-0.01	0.00	-0.00	-0.06
Boiled beans	0.11**	-0.02	0.07*	0.02	0.02	0.00	-0.07*	-0.02
Tofu	-0.00	-0.07^+^	0.02	-0.07	-0.07^+^	-0.06	0.00	-0.10*
Oranges	0.19**	0.03	0.15**	0.03	-	-	-0.12**	0.02
Fruits other than oranges	0.19**	0.09*	0.10**	0.04	-	-	-0.12**	-0.07^+^
Fruit juice	0.11**	0.03	0.07^+^	0.01	0.04	0.01	-0.09*	0.01

Analysis of 565-835males and 388-560 females.

** p<0.01, * p<0.05, + p<0.1

† : Crude correlation coefficients

‡ : Adjusted for age, smoking, drinking and serum concentration of total cholesterol.

§ : Adjusted for age, smoking, drinking, serum concentration of total cholesterol, and dietary intake of oranges, other fruits, green-leafy vegetables, carrots and squash, and tomatoes.

^#^Boiled fish paste, ^¶^Edible wild plants, ^† †^Food boiled down in soy sauce

The associations between individual carotenoid concentrations and food intake are shown in Table 3. Among males, intake of all green-yellow vegetables and fruits on the list were correlated with serum zeaxanthin/lutein concentration; intake of all fruits and vegetables, except tomatoes, was correlated with serum *α*- and *β*-carotene concentrations; intake of fruits was correlated with serum *β*-cryptoxanthin concentration; and intake of tomatoes and fruits was correlated with serum lycopene concentration. Among females, however, most of these correlations were not observed, except that intake of oranges was correlated with serum *β*-cryptoxanthin concentration and intake of tomatoes with serum lycopene concentration.

**Table 3.** Spearman’s correlation coefficients of specific dietary habits with carotenoids adjusted for age, smoking, and drinking.

	Zeaxanthin/ lutein	Cantha- xanthin	*β*-Crypto- xanthin	Total xantophylls	*α*-Carotene	*β*-Carotene	Lycopene	Total carotenes	Total carotenoids
Males									
Green-leafy vegetables	0.08*	0.05	0.03	0.07^+^	0.09**	0.06^+^	0.02	0.05	0.05
Carrots and squash	0.09**	0.00	0.01	0.06^+^	0.08*	0.08*	0.05	0.07*	0.06^+^
Tomatoes	0.09**	-0.00	-0.03	0.05	0.05	0.05	0.12**	0.08*	0.07*
Oranges	0.11**	0.09*	0.23**	0.17**	0.16**	0.14**	0.09*	0.14**	0.17**
Fruits other than oranges	0.10**	0.07^+^	0.16**	0.13**	0.12**	0.15**	0.08*	0.13**	0.13**

Eggs	0.14**	0.10**	0.05	0.13**	0.08*	0.09**	0.03	0.07*	0.11**
Milk	0.18**	0.13**	0.25**	0.22**	0.23**	0.25**	0.13**	0.24**	0.24**
*Sansai*^†^	-0.01	-0.09*	-0.15**	-0.06^+^	-0.09*	-0.13**	-0.10**	-0.14**	-0.11**

Females									
Green-leafy vegetables	0.04	-0.01	-0.00	0.04	-0.00	-0.00	-0.00	-0.00	0.01
Carrots and squash	0.03	-0.00	-0.00	0.01	0.00	-0.01	-0.01	-0.01	0.00
Tomatoes	0.06	0.05	-0.04	0.01	0.00	0.03	0.13**	0.07^+^	0.05
Oranges	0.02	0.10*	0.19**	0.11**	0.00	0.02	-0.13**	-0.03	0.04
Fruits other than oranges	0.06	0.00	0.09*	0.09^+^	0.02	0.03	-0.05	0.00	0.05

Eggs	0.06	0.02	0.00	0.03	-0.01	-0.02	-0.01	-0.02	-0.00
Milk	0.07^+^	0.06	0.08^+^	0.09*	0.06	0.06	-0.02	0.04	0.06
*Sansai*^†^	-0.04	-0.09*	-0.13**	-0.08^+^	-0.10*	-0.09^+^	-0.07	-0.09*	-0.10*

** p<0.01, * p<0.05, + p<0.1

† : Edible wild plants.

Smoking was negatively correlated with intake of eggs, milk, some oily foods, green-yellow vegetables, fruits, and beans in males, and with intake of miso soup, fried foods, fishes, some green-yellow vegetables, potatoes, and tofu in females (Table 2). Drinking in males was negatively correlated with ingestion of milk (*r_s_* = -0.10, p<0.01), boiled beans (*r_s_* = -0.10, p<0.01), oranges (*r_s_* = -0.09, p<0.05), and fruits other than oranges (*r_s_* = -0.10, p<0.01), but was positively correlated with intake of miso soup (*r_s_* = 0.07, p<0.05), liver (*r_s_* = 0.10, p<0.05), cheese (*r_s_* = 0.07, p<0.05), and sansai (*r_s_* = 0.13, p<0.01). In females, drinking was negatively correlated with intake of miso soup (*r_s_* = -0.09, p<0.05), carrots and squash (*r_s_* = -0.09, p<0.05), and tofu (*r_s_* = -0.09, p<0.05), and positively correlated with consumption of cheese (*r_s_* = 0.09, p<0.05), butter (*r_s_* = 0.13, p<0.01), margarine (*r_s_* = 0.12, p<0.01), and fruit juice (*r_s_* = 0.13, p<0.01).

## DISCUSSION

This study was primarily designed to validate the food frequency questionnaire and serum concentrations of carotenoids in the nested case-control studies as parts of the JACC Study.^8^^-^^10^ Controls of those nested case-control studies were thought to represent the general population of the JACC study, and adequate to the sample set of this study. However, discussion applied in a different situation may be limited; e.g., most subjects of the JACC Study lived in rural areas, where dietary habits may be different from those in urban areas; subjects who donated blood samples were around 30% of whole respondents to the baseline questionnaire survey, therefore, they may be more health-conscious.

Quantitative stability of carotenoids during long-term storage at -80°C is reviewed in the previous paper;^8^ briefly, decreases after 9 years of storage at -80°C were less than 15-20% for various carotenoids, and those serum levels measured in the JACC Study were comparable to those found in other studies in Japan.

In most comparisons of dietary intake of carotenoids and serum carotenoid concentrations, the amount of each individual carotenoid has been estimated using a semi-quantified food frequency questionnaire and a nutrient database.^4^^-^^6^ In the JACC study, however, a simple food frequency questionnaire (without portion size) for 32 food items was used. We therefore compared serum carotenoid concentrations with frequency of food intake using five frequency levels, which may explain why Spearman’s correlation coefficients were generally low (0.2 or less) in our study.

Serum carotenoid concentrations are lowered by smoking, a decrease attributed to the presence of *β*-unsaturated aldehydes and large numbers of free radicals in cigarette smoke.^2^ Alcohol drinking is also associated with decreased serum carotenoid concentrations.^2^ In this study, we found that, in male subjects, both smoking and drinking were negatively associated with the concentrations of most carotenoids. In female subjects, however, these associations were generally not observed, probably a much smaller percentage of females than males were smokers or drinkers. Other factors affecting serum carotenoid concentrations include those inhibiting the intestinal absorption of carotenoids or coexistent lipid-like substances,^2^ but these were generally not evaluated in this study. In this study, serum total cholesterol concentration was used to adjust carotenoid concentrations because lipoprotein fraction contains carotenoids and serum lipid fraction affects serum carotenoid concentrations.

Smoking and alcohol drinking are usually associated with other unfavorable lifestyle parameters, a finding confirmed in this study. We found that smoking was negatively associated with the intake of foods regarded in Japan as healthy, but the association between drinking and the intake of these foods was weaker. Serum concentration of total cholesterol could be correlated with serum carotenoid concentrations, as well as with dietary habits. These factors were therefore adjusted when evaluating the correlation between dietary habits and serum carotenoid concentrations.

After adjusting for smoking, drinking, and serum concentration of total cholesterol, the correlation between serum concentration of total carotenoids and intake of various foods was slightly changed in males. Among 192 nonsmoking males, there were positive correlations between serum concentration of total carotenoids and consumption of milk (*r_s_* = 0.24, p<0.01) and tomatoes (*r_s_* = 0.18, p<0.05), and weaker correlations between total carotenoids and intake of carrots and squash (*r_s_* = 0.12, p<0.1), oranges (*r_s_* = 0.09, p<0.1), and other fruits (*r_s_* = 0.04, p<0.1) (data not shown). Although milk contains some carotenoids, it is not regarded as directly associated with these nutrients. Nevertheless, among nonsmokers, intake of milk showed a higher association with serum carotenoid concentrations than did foods rich in carotenoids. These results suggest that the correlations adjusted for smoking, drinking, and total cholesterol in Table 2 may still contain residual confounding.

Further adjustment for intake of green-yellow vegetables and fruits, and for serum concentration of total cholesterol, was performed to determine the true association between serum carotenoid concentrations and foods, excluding those rich in carotenoids. Positive correlations of serum concentration of total carotenoids with intake of eggs and milk may be caused by confounding, although these foods contain some carotenoids. These foods are regarded as healthy, and their intake may be related to the intake of carotenoid-rich foods not surveyed in the questionnaire. The negative correlations between carotenoid concentrations and intake of *sansai* (edible wild plants) were not clearly interpreted, although individuals who eat large amounts of *sansai* may not eat sufficient vegetables.

Our finding of a lack of association between carotenoid concentrations and intake of green-yellow vegetables and fruit among female subjects was somewhat surprising, inasmuch as it is generally thought that women can recall their dietary habits more easily than men because women are more involved in food preparation than are men. In the studies of lung cancer in the JACC Study, consumption of green-yellow vegetables and fruits showed a clear protective effect against lung cancer deaths in males, but not in females.^12^ In contrast, high serum carotenoid concentrations were clearly associated with a decrease in the risk of lung cancer in both sexes^8^ and in females alone.^13^ These findings suggest that the food frequency questionnaire used in the JACC Study may not have reflected the dietary habits of women, especially in foods containing carotenoids.

There are several explanations for the poor correlations between food intake and serum carotenoid concentrations in females. First, each category in the questionnaire, such as green-leafy vegetables, carrots and squash, and fruits other than oranges, included the names of various kinds of foods. This may have led to some confusion and inter-subject differences in filling out the questionnaire. In addition, portion size was not surveyed. This explanation may be probable because specific relationships between tomatoes and lycopene, or oranges and *β*-cryptoxanthin were observed even in females. A second possibility is that subjects may have answered the questionnaire in such a way as to record what they thought their lifestyle should be, rather than what it was. The survey questionnaire was administered to the subjects to evaluate the association between lifestyle and illness, and the survey was linked with health checkups in some study areas. This possibility may be common among the subjects who were strongly interested in their health. Third, carotenoids are better absorbed when ingested with fat, and serum carotenoid concentrations correlate with serum lipid concentrations. Lipid metabolism may be unstable in middle-aged or elderly women; for example, serum cholesterol levels increase after menopause. Thus, in these women, serum carotenoid concentrations may be more affected by these lipid metabolic conditions than by dietary intake.

In conclusion, we have shown here a slight association between serum carotenoid concentrations and the intake of foods rich in carotenoids in males. Associations with intake of eggs and milk may be affected by confounding with foods rich in carotenoids that were not evaluated in the questionnaire. In females, the associations between serum carotenoid concentrations and intake of foods rich in carotenoids were scarce, suggesting that the food frequency questionnaire did not validly evaluate the dietary habits of females in the JACC Study. We observed several specific associations in both sexes, including those between serum *β*-cryptoxanthin concentration and consumption of oranges, and between serum lycopene concentration and ingestion of tomatoes.

## MEMBER OF LIST OF THE JACC STUDY GROUP

The present investigators involved, with the co-authorship of this paper, in the JACC Study and their affiliations are as follows: Dr. Akiko Tamakoshi (present chairman of the study group), Nagoya University Graduate School of Medicine; Dr. Mitsuru Mori, Sapporo Medical University School of Medicine; Dr. Yutaka Motohashi, Akita University School of Medicine; Dr. Ichiro Tsuji, Tohoku University Graduate School of Medicine; Dr. Yosikazu Nakamura, Jichi Medical School; Dr. Hiroyasu Iso, Institute of Community Medicine, University of Tsukuba; Dr. Haruo Mikami, Chiba Cancer Center; Dr. Yutaka Inaba, Juntendo University School of Medicine; Dr. Yoshiharu Hoshiyama, University of Human Arts and Sciences; Dr. Hiroshi Suzuki, Niigata University School of Medicine; Dr. Hiroyuki Shimizu, Gifu University School of Medicine; Dr. Hideaki Toyoshima, Nagoya University Graduate School of Medicine; Dr. Kenji Wakai, Aichi Cancer Center Research Institute; Dr. Shinkan Tokudome, Nagoya City University Graduate School of Medical Sciences; Dr. Yoshinori Ito, Fujita Health University School of Health Sciences; Dr. Shuji Hashimoto, Fujita Health University School of Medicine; Dr. Shogo Kikuchi, Aichi Medical University School of Medicine; Dr. Akio Koizumi, Graduate School of Medicine and Faculty of Medicine, Kyoto University; Dr. Takashi Kawamura, Kyoto University Center for Student Health; Dr. Yoshiyuki Watanabe, Kyoto Prefectural University of Medicine Graduate School of Medical Science; Dr. Tsuneharu Miki, Graduate School of Medical Science, Kyoto Prefectural University of Medicine; Dr. Chigusa Date, Faculty of Human Environmental Sciences, Mukogawa Women’s University ; Dr. Kiyomi Sakata, Wakayama Medical University; Dr. Takayuki Nose, Tottori University Faculty of Medicine; Dr. Norihiko Hayakawa, Research Institute for Radiation Biology and Medicine, Hiroshima University; Dr. Takesumi Yoshimura, Fukuoka Institute of Health and Environmental Sciences; Dr. Akira Shibata, Kurume University School of Medicine; Dr. Naoyuki Okamoto, Kanagawa Cancer Center; Dr. Hideo Shio, Moriyama Municipal Hospital; Dr. Yoshiyuki Ohno, Asahi Rosai Hospital; Dr. Tomoyuki Kitagawa, Cancer Institute of the Japanese Foundation for Cancer Research; Dr. Toshio Kuroki, Gifu University; and Dr. Kazuo Tajima, Aichi Cancer Center Research Institute.
